# Metabolomic Biomarkers in Gestational Diabetes Mellitus: A Review of the Evidence

**DOI:** 10.3390/ijms22115512

**Published:** 2021-05-24

**Authors:** Simon Alesi, Drishti Ghelani, Kate Rassie, Aya Mousa

**Affiliations:** 1 Monash Centre for Health Research and Implementation (MCHRI), School of Public Health and Preventive Medicine, Monash University, Melbourne 3168, Australia; simon.alesi@monash.edu (S.A.); drishti.ghelani@monash.edu (D.G.); kate.rassie@monash.edu (K.R.); 2 Department of Diabetes, Monash Health, Melbourne 3168, Australia

**Keywords:** gestational diabetes mellitus, metabolomics, biomarkers, metabolites, mass spectrometry, lipidomics

## Abstract

Gestational diabetes mellitus (GDM) is the fastest growing type of diabetes, affecting between 2 to 38% of pregnancies worldwide, varying considerably depending on diagnostic criteria used and sample population studied. Adverse obstetric outcomes include an increased risk of macrosomia, and higher rates of stillbirth, instrumental delivery, and birth trauma. Metabolomics, which is a platform used to analyse and characterise a large number of metabolites, is increasingly used to explore the pathophysiology of cardiometabolic conditions such as GDM. This review aims to summarise metabolomics studies in GDM (from inception to January 2021) in order to highlight prospective biomarkers for diagnosis, and to better understand the dysfunctional metabolic pathways underlying the condition. We found that the most commonly deranged pathways in GDM include amino acids (glutathione, alanine, valine, and serine), carbohydrates (2-hydroxybutyrate and 1,5-anhydroglucitol), and lipids (phosphatidylcholines and lysophosphatidylcholines). We also highlight the possibility of using certain metabolites as predictive markers for developing GDM, with the use of highly stratified modelling techniques. Limitations for metabolomic research are evaluated, and future directions for the field are suggested to aid in the integration of these findings into clinical practice.

## 1. Introduction

Gestational diabetes mellitus (GDM) is a common pregnancy complication, characterised by carbohydrate intolerance with onset or first recognition during pregnancy. It develops during pregnancy in women whose pancreatic function is insufficient to overcome the insulin resistance associated with the pregnant state, resulting in hyperglycaemia. GDM affects between 2 and 38% of pregnancies worldwide, with estimates of prevalence varying considerably based on diagnostic criteria used and sample population studied [1,2,3]. Risk factors for GDM include overweight and obesity, advanced maternal age, and a family history of any type of diabetes; and rates are escalating globally in parallel with the epidemics of obesity and type 2 diabetes mellitus (T2DM).

GDM places a heavy burden on patients and is associated with higher rates of adverse pregnancy outcomes, including pre-eclampsia, premature delivery, antenatal depression, instrumental or operative delivery and birth trauma [4,5,6]. Sudden intrauterine death can also occur, particularly in the setting of unrecognised GDM or poor glycaemic control. Babies born to mothers with GDM are often macrosomic with concomitant hypoglycaemia and jaundice [7,8]. Over the long term, children born to mothers with GDM are at an elevated risk of obesity and T2DM in later life [9]. The American Diabetes Association (ADA) formally classifies GDM as “diabetes first diagnosed in the second or third trimester of pregnancy that is not clearly overt (pre-existing type 1 or type 2) diabetes” [10]. GDM is typically diagnosed using an oral glucose tolerance test between 24 and 28 weeks of gestation. However, the International Association of Diabetes and Pregnancy Study Group (IADPSG) also recommends screening for overt diabetes at the first antenatal visit [11]. There is no clear consensus on which method should be used for this (fasting plasma glucose, random plasma glucose or HbA1c), nor whether it should be applied universally or only to high-risk population subgroups [11]. The diagnosis of “early GDM” thus remains controversial and methods are inconsistently applied. Hence, there remains a need to examine novel diagnostic biomarkers for GDM to facilitate early detection and treatment.

Metabolomics is a platform used to analyse and characterise a large number of metabolites [12]. Recently, metabolomics has been recognised as a potential tool to assess cardiometabolic conditions, including GDM, in the hope of improving screening and monitoring [13]. A previous review summarised metabolomic studies in GDM up to 2017; however, newly published studies have since emerged and may shed new insights on the potential use of these biomarkers in GDM and postpartum [14]. This review aims to outline some of the proposed metabolic derangements and mechanisms underlying the development of GDM. We also aim to highlight current and emerging metabolomic approaches to studying GDM and to discuss their utility in understanding these mechanisms. In doing so, metabolomics could present a unique avenue in the early detection of GDM, with the possibility of classifying risk in subsequent chronic disease among women and their progeny. This review is not systematic and was not intended to introduce new data or conclusions. Rather, we provide an overview of the literature by sourcing and evaluating findings from metabolomic studies published from inception to February 2021, that used a variety of biological matrices to identify potential biomarkers for GDM. Finally, we describe the limitations and future directions for research in this field, and the hurdles that must be overcome before integrating metabolomic approaches into clinical practice.

## 2. The Aetiology and Pathogenesis of GDM

The exact aetiology of GDM remains incompletely understood, but there are some putative mechanisms and risk factors that may help in understanding the progression of this condition.

### 2.1. Risk Factors for Gestational Diabetes

Epidemiological studies have outlined several risk factors for GDM, but these data are observational in nature and affected by residual confounding factors [15,16]. Moreover, there are different diagnostic criteria for GDM, making it challenging to draw comparisons between studies and countries [15,16]. Among the various risk factors associated with GDM, those that emerge consistently include pre-pregnancy obesity, pronounced gestational weight gain, Western diet, ethnicity, gene polymorphisms, advanced maternal age, pre-existing conditions related to insulin resistance (such as polycystic ovary syndrome), and a family history of diabetes [17,18,19].

### 2.2. Glucose Regulation during Healthy Pregnancy

**Figure 1:**
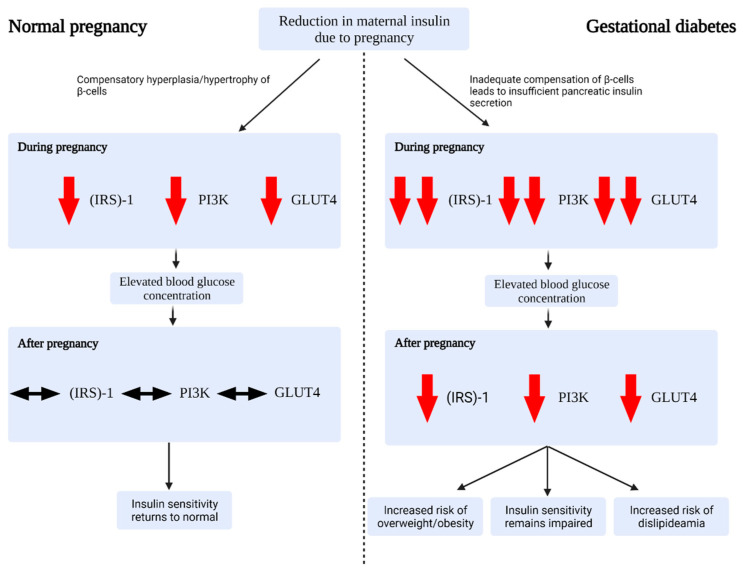
Proposed pathophysiology of gestational diabetes at different stages of pregnancy. During normal pregnancy, β-cells undergo compensatory hypertrophy/hyperplasia in order to meet the metabolic demands of pregnancy. A reduction in insulin sensitivity leads to a rise in glucose concentration. Following pregnancy, insulin sensitivity and blood glucose concentration return to normal. In gestational diabetes, β-cells inadequately compensate for the metabolic demands of pregnancy. The reduced insulin sensitivity results in hyperglycaemia. This is exacerbated by a precipitous reduction in insulin-receptor-substrate (IRS)-1, phosphoinositide 3-kinase (PI3K), and glucose transporter type 4 (GLUT4) expression. Following pregnancy, β-cells, blood glucose concentration, and insulin sensitivity may return to normal or remain impaired, resulting in an increased risk of developing obesity, a sustained impairment to insulin resistance, and dislipideamia.

During a normal pregnancy, the mother undergoes a series of adaptations in order to meet the physiological demands of the developing fetus. These adaptations include, but are not limited to, changes to the cardiovascular, renal, endocrine, and metabolic systems. One vital metabolic adaptation relates to insulin sensitivity (Figure 1) [17]: as pregnancy progresses, gradual rises in gestational hormones (including estrogen, progesterone, prolactin, cortisol, placental growth hormone and human placental lactogen) promote a state of persistent insulin resistance [17,20]. As such, blood glucose rises, allowing for ready transport of glucose to the fetus via the placenta [21]. In addition, this state of mild insulin resistance promotes endogenous glucose production and lipolysis, resulting in a further increase in blood glucose and a rise in free fatty acid (FFA) concentrations [17]. In order to maintain glucose tolerance, there is evidence for a parallel increase in maternal pancreatic islet cell mass secondary to β-cell hypertrophy and hyperplasia (enhancing insulin synthesis and glucose-stimulated secretion, and reducing the glucose-stimulation threshold) [22].

Ryan et al. [23] suggest that the importance of placental hormones in the development of insulin resistance is exemplified by the return of maternal insulin sensitivity to pre-pregnancy levels, which occurs in most women within hours post-delivery once placental hormones are washed from the maternal circulation.

### 2.3. Alterations in Gestational Diabetes

Outside of pregnancy, three distinct forms of diabetes are acknowledged. Type 1 diabetes (T1D) is a chronic autoimmune disease in which destruction of pancreatic β-cells causes insulin deficiency, leading to hyperglycaemia and a tendency to ketoacidosis [24]. T2DM has a basis in insulin resistance, although a reduction in insulin secretory capacity is observed over time [25]. “Secondary” causes of diabetes include genetic mutations, primary pancreatic diseases (pancreatitis, malignancy, and cystic fibrosis) and drug-induced forms [25,26].

Most women diagnosed with GDM will have a background of chronic insulin resistance (somewhat in keeping with the pathophysiology of T2DM), to which the normal insulin resistance of pregnancy is partially additive. This results in reduced glucose utilisation, increased glucose production and elevated FFA concentrations. If endocrine pancreatic function is insufficient, hyperglycaemia develops, resulting in the clinical picture of GDM (Figure 1) [17]. Specifically, there may be failures in the compensatory mechanisms of pancreatic β-cells, those cells found in the pancreatic islets that secrete insulin in response to a glucose load (Figure 1) [17]. In tandem with β-cell dysfunction, there appears to be a particular reduction in insulin sensitivity via altered expression of insulin receptor substrate (IRS)-1, phosphatidylinositol 3-kinase (PI3K), and glucose transporter 4 (GLUT4) [27,28,29]. A number of these metabolic and molecular adaptations persist throughout pregnancy and beyond, potentially leading to T2DM in predisposed women (Figure 1) [17,30].

## 3. Metabolomics as a Potential Tool to Investigate GDM

Metabolomics belongs to a branch of science that concentrates on characterising and quantifying biological molecules in the context of organic structure and function [31]. This branch is often termed ‘omics’, which includes a vast array of unique techniques for understanding multi-faceted conditions such as GDM [13,31].

Metabolomics is defined as the study of global metabolite profiles in a cell, tissue, or organism under a given set of conditions [12]. Metabolomics has several theoretical advantages over other ‘-omic’ approaches, making it more beneficial for assessing cardiometabolic conditions [12]. For instance, since the metabolome is the final downstream product of gene transcription, it is capable of integrating both epigenetic and genetic interactions involved in the progression of GDM [32,33,34]. Although the metabolome contains the smallest ‘omics’ domain, consisting of approximately 5000 metabolites, it is generally recognised as being more physically and chemically diverse than the other domains [32,33,35]. The metabolomic approach adopts the use of various separation and detection methods utilising high powered machinery, including gas chromatography, high-performance liquid chromatography, and mass spectrometry [36]. Lipidomics, which is a sub-set of metabolomics, aims to characterise and quantify lipid species, and is another important candidate for assessing metabolic conditions including GDM [37,38]. Given these advantages, metabolomics could provide unique insights into how multiple biomolecules interact with one another under certain conditions, to aid in the development of biomarkers for complex metabolic disorders such as GDM.

Recent literature has highlighted metabolomics as a prime candidate for evaluating the mechanisms underpinning GDM. Since metabolomics is equipped to characterise normal physiological as well as pathological states of biological systems, it is capable of identifying the subtle biochemical changes associated with endocrinopathies [35]. As such, metabolomics could be used to identify and isolate the novel biochemical disturbances underlying GDM. Relative advancements in the field of metabolomics have driven the formulation of the Mammalian Context Working Subgroups (MSI-MCWSG) of the Metabolomics Society to aid in the standardisation, curation, and communication of material from metabolomic studies [39]. Thus, this area of research is entering the forefront of clinical and diagnostic medicine, despite its relatively recent inception. To provide an up-to-date overview of the state of knowledge in this field, we outline below the recent metabolomic (and lipidomic) studies that have attempted to characterise GDM, from early diagnostic screening markers to the characterisation of deranged biochemical pathways.

## 4. The Metabolomic Profile of GDM: A Review of the Literature

GDM is a multi-faceted condition which involves a variety of deranged metabolic pathways including amino acids, carbohydrates, lipids, and purines. The details of these pathways, however, remain obscure. Table 1 highlights the main significantly altered metabolites in GDM, which will be explored and expanded upon further in the latter sections of the review. 

### 4.1. Potential Early Screening Diagnostic Markers and Models

Since GDM is a complex and multidimensional condition, it is important that prospective diagnostic markers are developed that are not confounded by other factors. Recent work has attempted to elucidate changes in the metabolome which occur both before and after the clinical diagnosis of GDM in early pregnancy, including prior to the incident dysregulation of blood glucose.

Nuclear magnetic resonance (NMR) spectroscopy may be conducted to analyse maternal sera and lipid extracts [40]. A study by Pinto et al. [40] of pre-diagnosis (gestational week 2–21) GDM, used this technique to show early metabolite changes in the maternal plasma and lipid extracted [40]. Pre-diagnosis GDM was associated with increases in plasma valine and pyruvate, with decreases in proline, urea, and 1,5-anhydroglucitol, compared to those who did not develop GDM. There were also minute decreases in glutamine, creatine, dimethyl sulfone, trimethyl amine N-oxide (TMAO), with increases in betaine and lactate and small increases in fatty acid and triglyceride levels [40]. However, post-diagnosis GDM showed a different and more pronounced set of alterations in metabolite levels [40]. After correcting for normal disparities in the plasma metabolomes due to late stage gestation, betaine, alanine, TMAO, methanol, and proline, were found to be significantly altered [40]. These changes indicate alterations in glycolysis, the tricarboxylic acid cycle (TCA), amino acid metabolism, urea cycle and lipid homeostasis. Additionally, some metabolites (trimethylamine (TMA)/TMAO, dimethyl sulfone and methanol) have been speculated to arise from dysregulated gut microbiota [46,47]. This is not unexpected, as low grade inflammation arising from dysregulated gut flora is also thought to contribute to T2DM disease pathology [48]. Pinto et al. [40] suggest that metabolite changes found in pre-diagnosis GDM are accentuated post-diagnosis, and that GDM prediction could be enhanced by exploiting multivariate changes in the metabolome rather than a set of univariate changes.

Hou et al. [41] utilised ultra-performance liquid chromatography-mass spectrometry (LC-MS), gas chromatography, and NMR on maternal serum (*n* = 131 GDM, and 138 controls) to develop a model that could accurately diagnose GDM using a combination of both clinical and metabolomic markers. It was found that there were general observed changes in FFAs, branched-chain amino acids (BCAAs), lipids, and organooxygen compounds, which differentiated both the control and GDM groups. They conducted receiver operating characteristic (ROC) analysis to assess the correlations of clinical data and metabolites with the risk of developing GDM. In general, the most discriminatory models for GDM risk prediction combined important biomarkers with key clinical parameters (such as BMI). Thus, as suggested by Hou et al. [39], in order to offer a more holistic view of metabolic perturbations in GDM, a multimarker approach for GDM diagnosis must be incorporated.

Pinto et al. [40] attempted to develop metabolic biomarkers of pre- and postdiagnosis GDM, with the use of NMR metabolomics of maternal blood and lipid extracts. They found that, whilst metabolomic changes seen in pre-diagnosis GDM appear intensified following diagnosis, glucose levels can be decreased following implementation of treatment strategies to manage the disease [40]. This glucose reduction was mostly associated with changes in lactate and pyruvate concentrations. However, there was no significant impact on the overall metabolomic profile (lipids, cholesterol, and amino acids), suggesting that while glucose levels are restored to normal, most of the underlying disease pathology persists [40]. This highlights that NMR metabolomics is capable of detecting the presence of the disease, independent of hyperglycaemia. Hence, NMR metabolomics could be used to develop a multi-metabolite biomarker, in cases where glucose tolerance tests alone are insufficient for clinical diagnosis. 

There is some evidence in support of a reduction in neonatal morbidity by achieving normoglycemia earlier in pregnancy with the adoption of early screening. For most healthy women, there is a lack of evidence linking early screening to improvements in neonatal outcomes [49]. However, high risk women are more likely to benefit from an early diagnosis of GDM. In a retrospective cohort study performed by Clarke et al. [49], women with an “early GDM” diagnosis (at an average of 17 weeks’ gestation) had better composite neonatal outcomes than their later-diagnosed peers, despite arguably representing a higher-risk cohort. The authors suggest that this may have resulted from earlier intervention and point to this approximate gestation as a beneficial screening point, as interventions can be made prior to the development of a functional fetal endocrine pancreas [50]. Since the average age of diagnosis of GDM is 24–28 weeks, there is a period of 7–10 weeks where the deleterious effects of the condition remain untreated [11,13]. This represents an important and compelling rationale to close this window via the development and improvement of early diagnostic criteria. Thus, a more detailed assessment of the metabolome in women with GDM, alongside clinical parameters in multivariate prediction models, could fill this gap in disease detection.

### 4.2. Amino Acid Profile in GDM

Plasma amino acids are often deranged in conditions pertaining to metabolic and oxidative stress [51]. Pathologic pregnancies, including GDM pregnancies, have been associated with increased oxidative stress, due to both increased circulating free radicals and/or a perturbation in antioxidant mechanisms [51]. Thus, the investigation of the amino acid profile in post-diagnosis GDM could reveal the potential causes of the condition.

In a study by O’Neill et al. [42], the metabolome of second trimester amniotic fluid (AF) samples from women diagnosed with GDM were profiled by assessing 459 known biochemicals via gas-chromatography/mass-spectrometry (GC-MS). The samples were then divided by sex, with male and female offspring. They found that the amino acid metabolites in general were significantly deranged in the AF of women with GDM. The most significant changes were in glucose, amino acid, glutathione, fatty acid, sphingolipid, and bile acid metabolism, and specific changes were identified based on the offspring sex. Significant changes in docosahexaenoic acid and arachidonic acid were also noted, and the authors suggested that sex-specific alterations in GDM maternofetal metabolism may begin to explain the sex-specific metabolic outcomes observed in offspring exposed to GDM in utero. This interesting possibility of a sexual dimorphism in metabolic risk is supported by a large population study by Ricart et al. [52] of 9270 women (with 4793 and 4477 male and female newborns, respectively). Here, maternal glucose tolerance status invariably influenced the risk of macrosomia in male but not female fetuses, and GDM predicted macrosomia in male fetuses exclusively [52]. Closer monitoring of glycaemic status may therefore be especially warranted in women carrying male fetuses; however, confirmation of this sex-specific variation awaits further study.

Scholtens et al. [43] used a GC-MS approach to evaluate broad-scale metabolic perturbations in hyperglycaemic mothers during pregnancy. They found that a variety of organic acids and amino acids, among others, were significantly altered. Although Enquobahrie et al. [53] also utilised a GC-MS approach to assess the metabolomic profile of GDM, they were mostly inconsistent with the findings of Scholtens et al. [43]. However, both of these studies found that alanine, valine, and serine were most commonly reported to be deranged [43,53]. At present, the significance of these amino acids in GDM pathophysiology is unclear, but researchers at the Joslin Diabetes Centre (Boston, MA, USA) posit that alanine may transiently reduce glucose levels by altering energy metabolism in the cell [54].

Aromatic amino acids (AAAs), including tyrosine and phenylalanine, have been shown to be significantly increased in large cohort studies of T2DM [55,56]. However, the relationship between AAAs and GDM pathogenesis may be more nuanced. Butte et al. [57] investigated protein metabolism in a small cohort of 16 Hispanic women (*n* = 8 insulin-treated GDM, and 8 controls) at late-stage gestation. They found elevated fasting and post-prandial AAAs at 32–36 weeks gestation in maternal plasma [57]. This contrasts with a more recent study which reported no change in AAA content at 30–33 weeks gestation in 25 women with GDM [58]. Other studies similarly found no change in specifically phenylalanine levels in maternal plasma at 30–39 or 37–41 weeks’ gestation [59,60]. These conflicting findings are unexpected, given that the role of AAAs (tyrosine and phenylalanine) in aberrant energy metabolism in insulin resistance has been well-established [57,61,62,63,64,65]. Increased levels of ketone bodies in GDM inhibits proteolysis and reduces oxidation of BCAAs and ketogenic amino acids in skeletal muscle, causing them to be released from skeletal muscle, and catabolised in the liver [58].

Studies investigating amino acid profiles in GDM, T2DM and non-diabetic pregnancies showed high plasma levels of arginine, glycine, and methionine in women with GDM [57,66]. The significance of arginine in GDM potentially stems from a dysregulation in the adenosine/L-arginine/nitric oxide (ALANO) pathway, which results in an accumulation in extracellular adenosine due to reduced uptake of adenosine into endothelial cells [67,68]. This may be the mechanism underlying vascular endothelial dysfunction in GDM. 

Tryptophan and purine metabolites in the urinary metabolome were investigated by Law et al. [69] in patients diagnosed with GDM using ultra-performance liquid chromatography. They found that tryptophan and purine metabolism was directly associated with GDM progression. Moreover, the kynurenine pathway, the metabolic pathway responsible for the production of nicotinamide adenine dinucleotide (NAD^+^) from tryptophan, was activated in participants with GDM before placental hormones or the fetoplacental unit could have produced any physiological effect [69]. Since dysregulation of this pathway is associated with genetic conditions, the authors postulate that GDM may be a predisposed condition in which a metabolically altered pre-diabetic state is fully realised during pregnancy [69]. This posits a challenge to conventional views of GDM pathogenesis, which place more emphasis on placental hormones as the primary contributor to insulin resistance in GDM. 

### 4.3. The Carbohydrate Profile in GDM

Alterations to carbohydrate metabolism during pregnancy are necessary in order to maintain a continuous supply of nutrients to the fetus via the placenta, despite intermittent maternal food intake [70]. 

In women with GDM, however, there may be a baseline of chronic insulin resistance (decreased insulin-stimulated glucose disposal and decreased inhibition of lipolysis) present pre-pregnancy. Gestation then adds another (transient, physiologic) source of insulin resistance, which exacerbates these defects. The ability of insulin to suppress lipolysis and hepatic glucose synthesis is reduced; and large post-prandial glucose excursions and impaired first-phase insulin secretion ensue. Catalano et al. [70] used hyperinsulinaemic-euglycaemic clamp studies to demonstrate these principles in women with GDM during late gestation. It would follow that assessing metabolites of carbohydrate metabolism would be beneficial in identifying potential biomarkers for pathogenesis. 

Spellacy et al. [71] measured blood glucose in AF obtained from 270 pregnant women. Unsurprisingly, they found that glucose levels were elevated in the GDM AF samples. For women with GDM, glucose concentration in AF represented maternal plasma glucose transported across the placenta via glucose transporters [71]. This was corroborated by Graca et al. [72] who reported increased glucose concentrations in second trimester AF of women later diagnosed with GDM, showing that mild glucose elevation is present in AF in the second trimester.

Gall et al. [44] attempted to assess early biomarkers of insulin resistance and glucose intolerance in a non-diabetic population. They performed multiple platform (ultra-high-performance liquid chromatography/gas chromatography) mass spectrometry, as well as non-targeted biochemical profiling, on a cohort of 399 nondiabetic participants (which represented a vast diversity in insulin sensitivity and glucose tolerance). They found that a series of glucose metabolism analytes were altered. This included increases in 2-hydroxybutyrate (AHB) and decreases in 1,5-anhydroglucitol (1,5-AG) and lactate. Increased AHB levels may be due to amino acid catabolism, activation of the glutathione stress pathway, or increased lipid peroxidation [44]. Prior studies have found that activation of these pathways correlated well with GDM incidence [73]. Increased AHB levels may also indicate mitochondrial dysfunction due to ineffective utilisation of propionyl-CoA in the TCA cycle. 

1,5-AG is a naturally occurring dietary monosaccharide. Being structurally similar to glucose, it competes with glucose for reabsorption in the renal tubules. High serum glucose prevents 1,5-AG renal reabsorption (as glucose is preferentially reabsorbed from the tubule filtrate), resulting in increased urinary excretion of 1,5-AG and thus lower serum levels. Decreased maternal serum levels of 1,5-AG indicate poor glycaemic control in the short-term in both pre-gestational diabetes and GDM (as in non-pregnant populations). This marker is in current clinical use in some diabetes treatment settings [66]. Wright et al. [66] demonstrated a statistically significant association between low 1, 5-AG levels during pregnancy and increased risk of diabetes-related pregnancy complications (specifically, large for gestational age infants and neonatal hypoglycaemia) in a cohort of women with GDM, T1D and T2DM. They suggested that serial 1, 5-AG measurements may help to guide decisions on the frequency of fetal ultrasound monitoring and on delivery planning, and proposed that 1,5-AG may provide unique information beyond that provided by HbA1c (and could be useful as an adjunct to self-monitoring of glucose for the ‘fine tuning’ of glycaemic control in pregnancies complicated by diabetes) [66]. 

Whilst the role of 1,5-AG in GDM is currently unknown, if the inverse relationship between blood glucose and 1,5-AG persists in pregnancy, then a reduction in 1,5-AG could be a useful indicator for early GDM diagnosis [66,74,75]. However, Hashimoto et al. [76] posits that due to a decrease in the threshold for glucose in the kidney during pregnancy, the glucose tolerance may not actually change, and glucosuria (glucose in the urine) may arise. Therefore, it is possible that a reduction in serum 1,5-AG does not necessarily reflect glycaemic control in pregnancy. Whilst 1,5-AG seems to be a promising target for biomarker identification, more thorough investigations are required to outline its role in GDM.

### 4.4. The Lipid Profile in GDM

Dyslipidaemia has long been implicated in glucose tolerance and obesity-related insulin resistance [38,77,78]. Thus, analysing lipids and how they function in GDM could help in understanding the pathophysiology of this condition. However, lipidomic studies in GDM are relatively scarce when compared to broader metabolomic studies [38]. This sub-section aims to highlight the recent lipidomic studies in GDM and the potential lipid biomarkers to consider, as well as how these findings could elevate our understanding of the aetiology of GDM. 

Rahman et al. [38] measured the plasma lipidome of 420 different metabolites at 8–13, 6–22, 24–29, and 34–37 weeks of gestation by GC-MS. They then used a linear mixed effects model to relate the different metabolites to the risk of developing GDM [38]. It was found that the mid-to-long carbon-chain glycerolipids were positively related to GDM, whilst the long carbon-chain cholesteryl esters were inversely related [38]. This finding has been previously reported [79]. The degree of this relationship was highly dependent on the week of gestation. Thus, lipid structure according to stage of pregnancy could be an important factor in determining GDM risk [38]. 

Anderson et al. [45] characterised the lipidome in three different groups; overt gestational diabetes (*n* = 18), hyperglycaemia but beneath the threshold for GDM (*n* = 45), and healthy controls (*n* = 43). They found that FFAs, phosphatidylcholines (PC) and lysophosphatidylcholines (LPC) had strong positive relationships with the risk of developing GDM [45]. This suggests that these lipids, particularly PCs, could contribute to disruptions in glucoregulatory mechanisms, immediately preceding hyperglycaemia [45].

Liangjian and colleagues [80] ran a prospective study of 817 (*n* = 200 discovery cohort and 617 validation cohort) pregnant women who provided serum samples at 28 weeks of gestation, and underwent an oral glucose tolerance test. Lipids were measured using a novel direct infusion mass spectrometry approach. Of the 13 different lipid species identified, 10 had significant associations with impaired glucose tolerance. The researchers highlighted five of the 10 lipids TG (50:1), TG (48:1), PC (32:1), PCae (40:3), and PCae (40:4) in the validation cohort that were independent of maternal age and body-mass index (BMI) [80]. This indicates that specific second trimester lipids may predict GDM independent of maternal age and weight status, which are known risk factors for the condition [80]. 

### 4.5. Prospective Diagnostic Markers for the Likelihood of Developing T2DM Postpartum

Following GDM pregnancy, there is a significant risk of lifetime progression to T2DM (20–50%, depending on the population studied) [81]. Underlying insulin resistance is likely to be the major mechanism, although some have suggested that irreversible damage to pancreatic β-cells from the severe glucose load during the gestational period may contribute [81]. Based on a 2015 observational study, Eades et al. [82] suggested that there is a viable time-window (<8 years postpartum) to prevent progression to T2DM. Thus, being able to identify at-risk patients using diagnostic biomarkers during this window of opportunity could enable targeted interventions and improved health outcomes.

Lai et al. [83] conducted a nested case-control study within a larger longitudinal study investigating metabolic imbalances in racially/ethnically diverse pregnant women diagnosed with GDM. At two years postpartum, they revisited samples from the early postpartum period (6–9 weeks), comparing serum of women who had developed T2DM (cases, *n* = 98) to those who had not (controls, *n* = 239). They were able to develop prediction models based on their findings, essentially discovering a distinct metabolic signature in the early postpartum period that predicted future T2DM. Metabolism of amino acids, arginine, proline, and BCAAs were elevated prior to T2DM with sustained or increased upregulation at follow-up visits in those who developed T2DM [83]. This corroborates previous studies, suggesting that amino acid metabolism plays a role in other insulin resistant states (e.g., polycystic ovary syndrome) and may influence insulin signalling and β-cell function [84]. Lipid metabolites, including sphingolipid concentrations, were also found to be reduced in those patients who progressed to T2DM [83]. This too is validated in the literature, where a reduction in sphingolipids is associated with the transition from GDM to T2DM, and more broadly the impairment of insulin secretion [81]. Considering that reduced sphingolipid biosynthesis impairs β-cell function, this puts forth a compelling argument that sphingolipid concentration contributes to the pathogenesis of T2DM postpartum.

Batchuluun et al. [85] profiled acylcarnitines in two diabetes cohorts (one group of women with GDM and a second group of women with historical GDM who developed glucose intolerance, T2DM, or returned to normoglycaemia within a 2-year follow up period). They found that short-chain acylcarnitines were implicated in the onset of T2DM following a GDM pregnancy [85]. This is not surprising given that they have previously been shown to negatively impact upon β-cell function [85]. However, the exact mechanisms in which this occurs are currently unknown and await further study [85]. 

Some lipidomic studies have found an association between plasma lipid profiles and the risk of developing T2DM in women who experienced GDM during pregnancy [81,86]. Lappas et al. [86] attempted to discern whether circulating lipid levels at 12 weeks postpartum following GDM pregnancy were related to the risk of developing T2DM postpartum. The plasma lipid profile (>300 lipids) of 104 women at 12 weeks postpartum following a GDM pregnancy were analysed using electrospray-ionisation tandem mass spectrometry [86]. All women had returned to normal glucose tolerance postpartum, but were then assessed for up to 10 years for the development of incident T2DM [86]. Over a median follow-up period of 8.5 years, 20% (*n* = 21) of the women developed T2DM. In this cohort, the three lipid species that were most associated with the risk of developing T2DM were the cholesteryl ester species, the alkenyl phosphatidylethanolamine species, and the phosphatidylserine species [86]. The authors suggested that these lipid species could be used as biomarkers to predict the development of T2DM after GDM, such that preventive and/or treatment measures could be employed to slow or abrogate disease progression [86]. 

## 5. Limitations and Future Directions

This preliminary data, albeit sparse, highlights the potential utility of metabolomics in the assessment, early screening, and treatment of GDM. However, the metabolomic approach has important limitations that should be considered in the context of these findings. The metabolome is influenced by intrinsic (gender, ethnicity, epigenetics, and genetic mutations) and extrinsic (environment, stress, and diet) factors. Thus, minute intra-individual differences are exacerbated, significantly affecting the metabolomic profile. The lack of high-powered metabolomic studies, particularly with respect to GDM, further amplifies this problem. In addition, metabolomics is not equipped to account for divergences in participant demographics or co-morbid conditions that influence the metabolome. Of note, there was low repeatability between findings in the outlined metabolomic studies. It is possible that the inherent heterogeneity of the metabolome and the current inability to control for extraneous variables could be propagating these disparate outcomes. 

The selected studies for this review adopted different diagnostic criteria for GDM. The diagnostic criteria utilised varied from the ADA, World Health Organisation (WHO), IADPSG, Carpenter and Coustan (prior to 2011), as well as others. This makes it particularly challenging to make direct comparisons between the selected studies, as the diagnostic criteria for GDM varies considerably. As scientific inquiry progresses, utilising metabolomics and other approaches, a standardised diagnosis of GDM could be developed on which future research is based. This would allow for appropriate comparison between studies in terms of their metabolic profile. 

Whilst LC-MS-based metabolomic approaches were most common in the reviewed literature, there were other detection methods that were used to analyse metabolites. Therefore, it is possible that the lack of reproducibility of these findings could be attributed to a variation in detection methods. To further complicate matters, each metabolomic technique (from LC-MS to GC-MS) has a separate database for biomarker identification [87]. This could create difficulties in comparing biomarker identity between studies that used different metabolomic techniques [87]. Unfortunately, the only way to remedy this problem is to continuously revise these databases so that they are up-to-date with the research [87]. 

As suggested, future studies should focus on addressing the above limitations prior to considering the application of these findings into clinical practice. This will allow metabolomics to be integrated with other ‘omic’ disciplines, which will improve the specificity of the analysis. While observational studies can offer large datasets with nationally representative samples, metabolomic research should also incorporate controlled clinical trials, which are better equipped to control for key confounders (diet, ethnicity etc) and, with sufficiently powered analyses, can determine potential causation.

## 6. Conclusions

In this review of the literature, we have discussed novel biomarkers for GDM assessed via metabolomics techniques, with the aim of outlining potential predictive strategies and/or characterising the metabolic disturbances present in GDM. Many studies point to the possibility of predicting GDM by combining clinical biomarkers with multivariate modelling (often also incorporating key clinical variables and known risk factors). Whilst many different metabolic pathways have been shown to be deranged in GDM, the aetiology of the condition remains poorly understood and no single biomarker candidate has yet demonstrated clinical utility. Despite this, key pathways of significance for future research in the pathogenesis of GDM include amino acid metabolism, carbohydrate metabolism, purines, and lipids. In relation to prospective clinical biomarkers for the risk of postpartum progression to T2DM following GDM pregnancy, current research suggests that BCAAs, acylcarnitines, and specific lipid species or classes (e.g., sphingolipids) are implicated in this conversion. Whilst these findings are promising, there are many limitations that must be overcome prior to their integration into clinical practice. Therefore, whilst metabolomics has provided some unique insights into the pathogenesis and aetiology of GDM, further research is needed before this technique can meet the necessary thresholds to be considered for clinical application.

## Figures and Tables

**Table 1:** Summary of studies highlighting altered metabolites in GDM and insulin resistance in pregnancy.

Author, Year [Ref]	Population	Metabolomic Platform(s)	Metabolic Medium	Main Altered Metabolites in GDM
**Potential Early Screening Markers**				
Pinto, et al., 2015 [40]	Pre-diagnosis GDM (2–21 weeks gestation) who later developed GDM (*n* = 41–93)	NMR	Maternal plasma and lipid extracts	Pre-diagnosis GDM showed increases in plasma valine and pyruvate, with decreases in proline and urea
Hou, et al., 2018 [41]	*n* = 131 women with GDM and 138 controls	UHPLC-MS, GC, NMR	Maternal serum	Perturbations in free fatty acids, branched chain amino acids, and organooxygen compounds in the GDM group
**Amino Acids**				
O’Neill, et al., 2018 [42]	*n* = 20 women with second trimester GDM diagnosis	GC-MS	Amniotic fluid	Glutathione was increased, which may be related to increased lipid peroxidation in GDM
Scholtens, et al., 2014 [43]	*n* = 67 high FPG; *n* = 50 low FPG at ~28 weeks gestation	GC-MS	Fasting serum	Alanine, valine, and serine were most commonly deranged
**Carbohydrates**				
Gall, et al., 2010 [44]	*n* = 399 non-diabetic pregnant women with varying degrees of insulin sensitivity	UHPLC/ GC-MS	Fasting plasma samples	Increases in 2-hydroxybutyrate (AHB), and decreases in 1,5-anhydroglucitol and lactate were associated with reduced insulin sensitivity
**Lipids**				
Rahman, et al., 2018 [38]	*n* = 107 women with GDM, and 214 without GDM	GC-MS	Plasma	Mid-to-long carbon chain glycerolipids were positively related to GDM
Anderson, et al., 2014 [45]	Women with overt GDM (*n* = 18); hyperglycaemia (*n* = 45); or healthy controls (*n* = 43)	UPLC-MS	Fasting serum	Phosphatidylcholines and lysophosphatidylcholines had strong positive relationships with GDM

FPG, fasting plasma glucose; GDM, gestational diabetes mellitus; GC-MS, gas chromatography-mass spectrometry NMR, nuclear magnetic resonance; UHPLC-MS/UPLC-MS, ultra-performance/ultra-high performance liquid chromatography-mass spectrometry.
